# Takotsubo (stress) cardiomyopathy induced by acute asthma exacerbation in elderly woman

**DOI:** 10.1002/ccr3.7074

**Published:** 2023-03-08

**Authors:** Khaled Al Khodari, Raad Alhaj Tahtouh, Waleed K. Abdullatef, Mouaz Al Khodari

**Affiliations:** ^1^ Cardiology Department Hamad Medical Corporation (HMC) Doha Qatar; ^2^ Internal Medicine Department Hamad Medical Corporation (HMC) Doha Qatar; ^3^ Medical School Syrian Private University Damascus Syria

**Keywords:** asthma exacerbation, left ventricular dysfunction, stress cardiomyopathy, sympathomimetic drugs, takotsubo cardiomyopathy

## Abstract

A 73‐year‐old patient who was admitted secondary to acute asthma exacerbation that required frequent salbutamol and adrenaline nebulization. Takotsubo cardiomyopathy (TTC) was diagnosed after the new onset of chest pain with modest troponin elevation and normal coronary angiogram. Low ejection fraction and apical akinesia were completely resolved after her symptoms got improved.

## Abstract 2

Physical stressors, like acute illnesses, can trigger takotsubo cardiomyopathy (TTC). Excessive exposure to inhaled sympathomimetic agents, such as adrenaline or salbutamol, increase the risk of takotsubo cardiomyopathy (TTC). Tight asthma control is important to minimize the risk of severe complications related to the disease itself and its management.

## 1 INTRODUCTION

Takotsubo cardiomyopathy (TTC), also called stress cardiomyopathy or broken heart syndrome, is a reversible myocardial disease that is characterized by brief systolic and diastolic dysfunction and regional wall motion abnormalities that extend beyond the territory of single coronary artery distribution.^1^ It affects, most likely, postmenopausal women, and the exact prevalence is unknown because TTC is most likely misdiagnosed as an acute coronary syndrome. However, it accounts for 1%–2% of all suspected cases of the acute coronary syndrome.^2^

The pathophysiology of takotsubo cardiomyopathy is not well established, but it is associated with morphological changes in myocardium secondary to catecholamine surge, in response to stress exposure, which leads to direct myocardial toxicity and diffuse microcirculatory dysfunction.^3^

Emotional and/or physical stressors can trigger TTC. Personal or family crisis events, like the death of a loved one, are the most common emotional stressors; however, acute noncardiac disease, trauma, or surgery are the typical reported physical triggers.^4^

Asthma exacerbation is considered a potential trigger of stress‐induced cardiomyopathy as in our patient who had acute exacerbation that required hospital admission and frequent puffs of albuterol (beta‐2 agonist) and adrenaline nebulization which induced acute transient drop of ejection fraction and apical akinesia that improved completely with effective asthma management.

## 2 CASE PRESENTATION

A 73‐year‐old female patient with the past history of uncontrolled asthma with frequent exacerbations requiring recurrent hospital admissions. She was on fluticasone/salmeterol puffs twice daily and albuterol as needed, but she was not compliant with her medications. She presented to emergency department (ED) complaining of 1 week's history of dry cough and worsening shortness of breath (SOB). There was no fever, flu‐like symptoms, chest pain, or palpitation. In ED, she looked anxious, and she was tachypneic with respiratory rate (RR) around 27 per minute and requiring 5 L of oxygen therapy to keep saturation around 95% upon initial assessment. Her temperature, heart rate, and blood pressure were within normal range. Upon chest auscultation, bilateral diffuse wheezing with prolonged expiration discovered was detected. The partial pressure of oxygen (PO2), carbon dioxide (PCO2), and PH were 58 mmHg, 52 mmHg, and 7.34, respectively, by arterial blood gas (ABG) analysis. Complete blood count (CBC), renal and liver function tests, and inflammatory markers were normal. There was neither congestion nor consolidation on the chest X‐ray.

The patient was started on back‐to‐back albuterol nebulization and received both adrenaline nebulization and intravenous (IV) hydrocortisone. One dose of IV magnesium sulfate was given. Her SOB Settled down; but 3 h later, the patient complained of central chest pain that is associated with palpitation. Vital signs were stable. Electrocardiogram (ECG) revealed sinus rhythm and old T wave inversion in lead AVL. Serum high sensitivity troponin T level was 400 ng/L, the normal reference range is less than 14 ng/L, which dropped to 59 ng/L upon follow‐up 4 days later.

The initial impression was non‐ST‐elevation myocardial infarction (NSTEMI); therefore, the patient was started on dual antiplatelet therapy and IV heparin infusion. Echocardiography revealed moderately reduced left ventricular (LV) systolic function (Ejection fraction = 32%) with dilated akinetic apex, Video S1A. The coronary angiogram was completely normal.

The patient clinical status remained stable over the next few days in terms of oxygen requirements, chest pain, and SOB. The patient is claustrophobic and totally refused Cardiovascular magnetic resonance (CMR). Repeated echocardiography exhibited complete resolution of LV systolic dysfunction and apical akinesia, Video S1B. Thus, the diagnosis of takotsubo cardiomyopathy (TTC) was made. She was discharged in stable condition after optimization of her anti‐asthma medications; she is educated well about the importance of compliance to medications, and montelukast 10 mg was added. Five milligrams of lisinopril and 1.25 mg of bisoprolol, which she tolerated well, were included in her discharge medications. Three months later, the patient was asymptomatic and complaint of medications. Repeated echocardiography was normal.

## 3 DISCUSSION

According to the modified Mayo Clinic criteria, all four criteria must meet to make the diagnosis of TTC: (1) transient left ventricular hypokinesia, akinesia, or dyskinesia of mid‐segments, with or without apical involvement, that extends beyond the distribution of single coronary artery, with or without an identifiable stressor; (2) the absence of obstructive coronary artery disease or acute plaque rupture by coronary angiogram; (3) new ECG abnormalities or modest troponin elevation; (4) exclusion of pheochromocytoma or myocarditis.^5^

Recognition of stress cardiomyopathy in patients with acute respiratory illness is challenging because both have the same clinical presentation in terms of chest pain and shortness of breath. A systematic review of 38 patients with acute pulmonary disease and TTC concluded that a relationship between both illnesses is present and patients with severe respiratory diseases are at increased risk of evolving TTC.^6^ It revealed that not only asthmatic patients have such risk, but also those with other acute pulmonary problems, like patients with chronic obstructive pulmonary disease (COPD), pulmonary embolism, severe pneumonia, or even patients with lung cancer who require invasive diagnostic or therapeutic procedures.^6^

The possibility of TTC in the course of acute asthma, and other severe respiratory diseases, is believed to be related to excessive myocardial exposure to catecholamines secondary to the stress related to the disease itself and frequent use of sympathomimetic drugs, like beta‐2 agonist or adrenaline, for symptom relief and disease control. Moreover, hypoxia, hypercapnia, and acidosis, which might complicate the pulmonary disease, might increase myocardial catecholamine release and sympathetic nerve stimulation.^7^

In addition to control the source of stress, the management of TTC is mainly supportive to treat the symptoms and to minimize the complications. In cases when pulmonary congestion is present, diuretics plus vasodilators, like nitroglycerin, can be given. For those with hypotension and cardiogenic shock, mechanical circulatory support devices can be used. Inotropes might be a good option if LV outflow tract obstruction is ruled out by echocardiography. Beta‐blockers can be added in stable patients to protect against arrhythmias.^8^

The prognosis of TTC is generally good with most patients achieve full recovery within a few weeks. However, stress cardiomyopathy has an in‐hospital mortality rate of around 5%.^8^ The reported recurrence rate of TCC is 2%–4% per year.^9^ The effect of beta‐blockers on recurrence prevention has not been proven yet, but they may play a role in selected patients, such as those with persistent symptoms or previous history of recurrent TTC.^10^

## 4 CONCLUSION

Clinicians should be aware of the possible association between acute asthma exacerbation and TTC in order to optimize asthma management to prevent acute exacerbations and overuse of sympathomimetic drugs which may increase the risk of stress cardiomyopathy.

## AUTHOR CONTRIBUTIONS

**Khaled Al Khodari:** Formal analysis; investigation; supervision; writing – original draft; writing – review and editing. **Raad Alhaj Tahtouh:** Investigation; writing – original draft. **Waleed K. Abdullatef:** Investigation; supervision; writing – review and editing. **Mouaz Al Khodari:** Investigation; writing – original draft.

## CONFLICT OF INTEREST STATEMENT

The authors have no relationships relevant to the contents of the paper to disclose.

## FUNDING INFORMATION

None.

## ETHICAL APPROVAL

Ethical approval to report this case was obtained from Hamad Medical Corporation (HMC) Ethics Committee; ID: MRC‐04‐22‐361.

## CONSENT

Written informed consent was obtained from the patient to publish this report in accordance with the journal's patient consent policy.

## Supporting information


Video S1

## Data Availability

The data that support the findings of this study are available from the corresponding author, Khaled Al Khodari, upon reasonable request.

## References

[1] Akashi YJ , Goldstein DS , Barbaro G , Ueyama T . Takotsubo cardiomyopathy: a new form of acute, reversible heart failure. Circulation. 2008;118(25):2754‐2762. doi:10.1161/CIRCULATIONAHA.108.767012 19106400 PMC4893309

[2] Gianni M , Dentali F , Grandi AM , Sumner G , Hiralal R , Lonn E . Apical ballooning syndrome or takotsubo cardiomyopathy: a systematic review. Eur Heart J. 2006;27(13):1523‐1529. doi:10.1093/eurheartj/ehl032 16720686

[3] Nef HM , Möllmann H , Kostin S , et al. Tako‐Tsubo cardiomyopathy: intraindividual structural analysis in the acute phase and after functional recovery. Eur Heart J. 2007;28(20):2456‐2464. doi:10.1093/eurheartj/ehl570 17395683

[4] Sharkey SW , Windenburg DC , Lesser JR , et al. Natural history and expansive clinical profile of stress (tako‐tsubo) cardiomyopathy. J Am Coll Cardiol. 2010;55(4):333‐341. doi:10.1016/j.jacc.2009.08.057 20117439

[5] Scantlebury DC , Prasad A . Diagnosis of takotsubo cardiomyopathy – Mayo Clinic criteria. Circ J. 2014;78(9):2129‐2139. doi:10.1253/circj.CJ-14-0859 25131525

[6] Manfredini R , Fabbian F , Giorgi AD , et al. Heart and lung, a dangerous liaison‐Tako‐tsubo cardiomyopathy and respiratory diseases: a systematic review. World J Cardiol. 2014;6(5):338‐344. doi:10.4330/wjc.v6.i5.338 24944763 PMC4062124

[7] Kotsiou OS , Douras A , Makris D , Mpaka N , Gourgoulianis KI . Takotsubo cardiomyopathy: a known unknown foe of asthma. J Asthma. 2017;54(8):880‐886. doi:10.1080/02770903.2016.1276586 28055270

[8] Lyon AR , Citro R , Schneider B , et al. Pathophysiology of Takotsubo syndrome: JACC state‐of‐the‐art review. J Am Coll Cardiol. 2021;77(7):902‐921. doi:10.1016/j.jacc.2020.10.060 33602474

[9] Akashi YJ , Nef HM , Lyon AR . Epidemiology and pathophysiology of Takotsubo syndrome. Nat Rev Cardiol. 2015;12(7):387‐397. doi:10.1038/nrcardio.2015.39 25855605

[10] Lyon AR , Bossone E , Schneider B , et al. Current state of knowledge on Takotsubo syndrome: a position statement from the Taskforce on Takotsubo Syndrome of the Heart Failure Association of the European Society of Cardiology. Eur J Heart Fail. 2016;18(1):8‐27. doi:10.1002/ejhf.424 26548803

